# POSITIVE SELF-PERCEPTIONS OF AGING AS PREDICTORS OF COVID-RELATED PREVENTIVE BEHAVIOR AND RESILIENCE

**DOI:** 10.1093/geroni/igac059.376

**Published:** 2022-12-20

**Authors:** Hannah Giasson, William Chopik, Alejandro Carrillo

**Affiliations:** Arizona State University, Tempe, Arizona, United States; Michigan State University, East Lansing, Michigan, United States; Michigan State University, East Lansing, Michigan, United States

## Abstract

Individuals have faced extraordinary challenges throughout the COVID-19 pandemic. Psychosocial strengths may promote individuals’ resilience during this time. Positive self-perceptions of aging (SPA) have been found to predict a variety of health and well-being indicators. We examined SPA as a predictor of COVID-19-related behavior, adaptation, and resilience in a sample of 3,620 adults (Mage=65.88; 61.1% women; 65.4% white) from the 2016 and 2020 waves of the Health and Retirement Study. Linear regression results revealed that more positive SPA in 2016 was associated with more preventative health behavior (β=.03, p=.04), a higher likelihood of staying at home (β=.07, p<.001), less worry (β=-.27, p<.001), less stress (β=-.24, p<.001), less loneliness (β=-.27, p<.001), and greater resilience (β=.20, p<.001) during the first year of the pandemic (2020). Associations held after controlling for demographic covariates. Findings support SPA theories, suggesting linkages between SPA and adaptive behaviors and outcomes in the face of external challenges.

